# Modeling Human Natural Killer Cell Development in the Era of Innate Lymphoid Cells

**DOI:** 10.3389/fimmu.2017.00360

**Published:** 2017-03-27

**Authors:** Steven D. Scoville, Aharon G. Freud, Michael A. Caligiuri

**Affiliations:** ^1^Biomedical Sciences Graduate Program, Medical Scientist Training Program, The James Cancer Hospital and Solove Research Institute, The Ohio State University, Columbus, OH, USA; ^2^Comprehensive Cancer Center, The James Cancer Hospital and Solove Research Institute, The Ohio State University, Columbus, OH, USA; ^3^Department of Pathology, The James Cancer Hospital and Solove Research Institute, The Ohio State University, Columbus, OH, USA; ^4^Division of Hematology and Oncology, Department of Internal Medicine, The James Cancer Hospital and Solove Research Institute, The Ohio State University, Columbus, OH, USA

**Keywords:** natural killer, innate lymphoid cells, development, secondary lymphoid tissues, human lymphopoiesis

## Abstract

Decades after the discovery of natural killer (NK) cells, their developmental pathways in mice and humans have not yet been completely deciphered. Accumulating evidence indicates that NK cells can develop in multiple tissues throughout the body. Moreover, detailed and comprehensive models of NK cell development were proposed soon after the turn of the century. However, with the recent identification and characterization of other subtypes of innate lymphoid cells (ILCs), which show some overlapping functional and phenotypic features with NK cell developmental intermediates, the distinct stages through which human NK cells develop from early hematopoietic progenitor cells remain unclear. Thus, there is a need to reassess and refine older models of NK cell development in the context of new data and in the era of ILCs. Our group has focused on elucidating the developmental pathway of human NK cells in secondary lymphoid tissues (SLTs), including tonsils and lymph nodes. Here, we provide an update of recent progress that has been made with regard to human NK cell development in SLTs, and we discuss these new findings in the context of contemporary models of ILC development.

## Introduction

Natural killer (NK) cells belong to the family of innate lymphoid cells (ILCs) whose common features include reliance on the transcription factor ID2 for development and rapid elaboration of effector function in response to microbial products, cytokine stimulation, and contact with other leukocytes (1). ILCs share phenotypic and functional features with T cells, yet, ILCs lack expression of markers specific for other leukocytes, including antigen-specific T and B cell receptors (i.e., CD3/TCR and CD19/BCR). In 2013, Spits et al. published a proposal for ILC nomenclature and classification, including the designation of three major groups according to functional and phenotypic characteristics (2). Group 1 ILCs, which express the transcription factor T-BET and produce the T helper cell type 1 (Th1)-associated cytokine interferon gamma (IFN-γ), include NK cells as well as functionally distinct “ILC1s.” NK cells are unique in their expression of the transcription factor EOMES and in their ability to recognize and destroy virally infected and malignantly transformed cells that have downregulated major histocompatibility (MHC) class I molecules and/or upregulated stress-induced molecules (3, 4). Group 2 ILCs or “ILC2s” produce Th2-associated cytokines, such as interleukin (IL)-5 and IL-13; they express the transcription factors BCL11B, GATA-3, and RORα; and they are involved in many processes, including fat metabolism, allergy, and protection against parasites (5). Group 3 ILCs share features with Th17 cells, including expression of the transcription factors AHR and RORγt and production of IL-17 and IL-22. Group 3 ILCs consist of “ILC3s” as well as lymphoid tissue inducer cells whose roles include the formation and restoration of lymphoid tissues following infection (6). We refer the readers to other excellent comprehensive reviews exploring the development, transcriptional regulation, and diverse roles of ILCs in physiology and disease (1, 7–12).

As mentioned above, ILCs are identified as “lineage” (Lin) negative lymphocytes, lacking expression of surface markers more specifically expressed on T cells (CD3, CD5, TCR), B cells (CD19, CD20, BCR), myelomonocytic cells (CD14, CD15, CD36), and dendritic cells (DCs) (CD116, CD123, CD303). In humans, all non-NK ILCs express CD127 (IL-7Rα) and CD161 (NKRP1A) (13–16), and they may be further distinguished according to the expression of other subset-associated surface antigens including CXCR3, CD294 (CRTH2), and CD117 (c-Kit) for ILC1s, ILC2s, and ILC3s, respectively (10, 17). Human NK cells can also express the pan-ILC markers CD127 and CD161 (18–20), but NK cells are typically distinguished by their surface expression of CD16 (FcγRIIIA), CD94/NKG2 heterodimers, killer immunoglobulin-like receptors (KIRs), NKG2D, and NKp80 (21). Two subsets of human peripheral blood (PB) NK cells can be distinguished according to their relative expression of the pan-NK cell surface marker, CD56 (NCAM-1): “CD56^bright^” and “CD56^dim^” (22, 23). CD56^bright^ human NK cells express high levels of CD62L (l-selectin) and CD94 but absent or low levels of CD16 and KIRs; they predominate in secondary lymphoid tissues (SLTs) such as lymph nodes (LNs) and tonsils; they show low baseline perforin expression and cytotoxic activity *ex vivo*; and they rapidly produce cytokines, including IFN-γ, following stimulation by monocyte-derived cytokines (“monokines”), such as IL-12, IL-15, and IL-18 (22). In contrast, CD56^dim^ NK cells are CD16^hi^ and express more KIRs but less CD94 and CD62L; they predominate in PB; they show high baseline perforin expression and cytotoxicity against MHC class I negative target cells; and they preferentially produce cytokines in response to direct target cell interactions rather than *via* monokine stimulation (3). While the developmental relationship between these human NK cell subsets has not been definitively established, evidence suggests that CD56^bright^ NK cells represent immediate physiologic precursors of CD56^dim^ NK cells (19, 24–29). Alternative hypotheses include that CD56^bright^ NK cells represent activated NK cells *in vivo* and/or that PB NK cell subsets derive from distinct hematopoietic progenitor cells (HPCs) and developmental pathways (22, 30–33). Recent published data from Dunbar and colleagues suggest that the latter may be the case in rhesus macaques (34).

## Human NK Cell Development in SLTs

**Figure 1:**
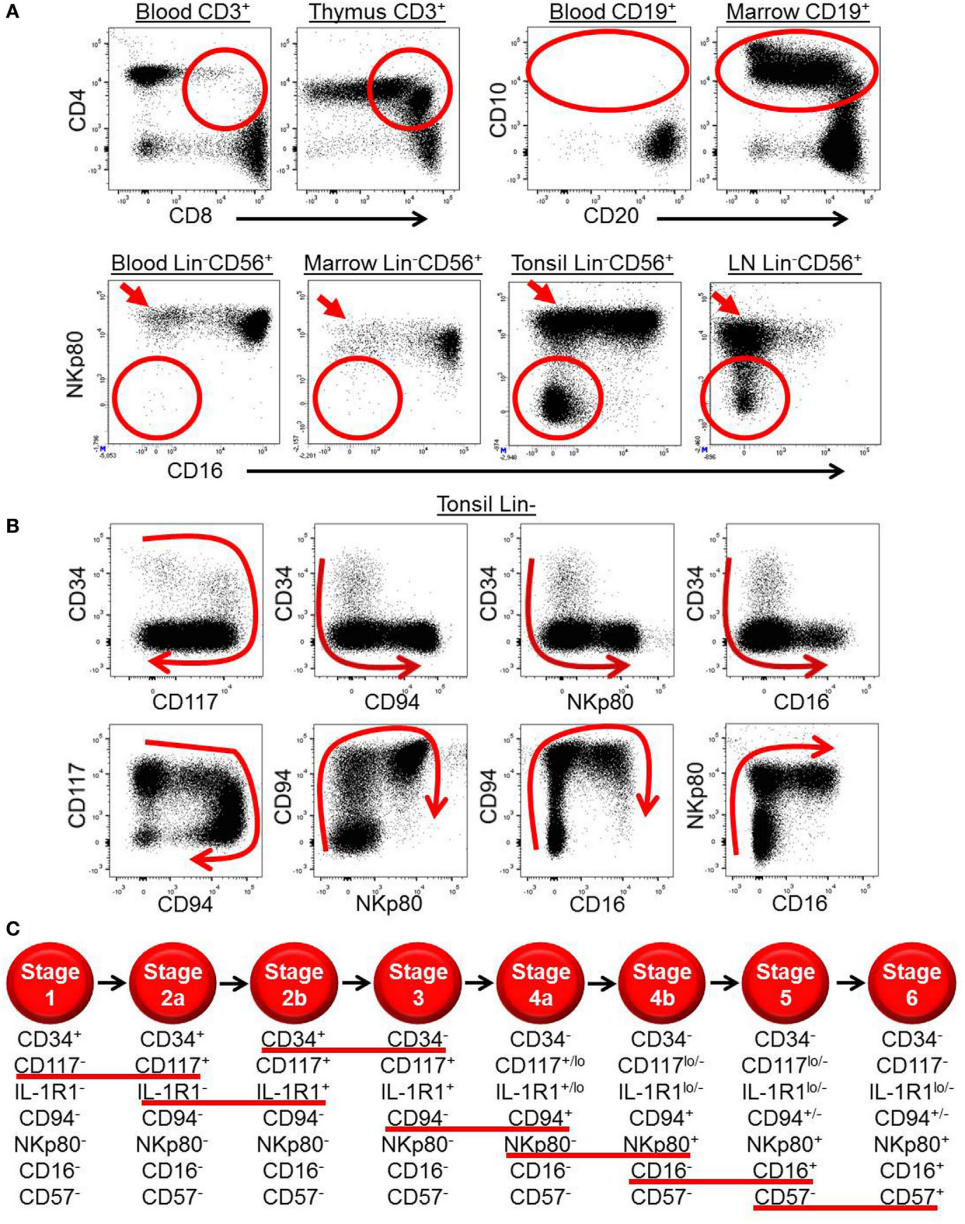
***Ex vivo* patterns of surface antigen expression support a model of human natural killer (NK) cell development in secondary lymphoid tissues (SLTs)**. **(A)** *Ex vivo* immunophenotypic analyses of CD3^+^ cells (top row, left plots), CD19^+^ cells (top row, right plots), and Lin^−^CD56^+^ cells (bottom row) in the indicated tissues demonstrate how immature T, B, and NK cell developmental intermediates (designated by the red circles and ovals) are naturally enriched in the thymus, bone marrow, and SLTs, respectively. Of note, the SLT populations designated by the red circles in the bottom row also likely contain some ILC3s, which can express CD56 (14). The red arrows in the bottom row highlight the relative enrichment of stage 4b CD56^bright^NKp80^+^CD16^−^ NK cells in SLTs. **(B)** Immunophenotypic analysis of Lin^−^ ILCs in human tonsil demonstrating the two-way patterns of CD34, CD117, CD94, NKp80, and CD16 expression as they relate to one another. The red arrows depict the putative directions of progressive NK cell development in SLTs. **(C)** Schematic representation of the proposed stages of human NK cell development in SLTs. The stages are defined according to the differential expression of CD34, CD117, interleukin (IL)-1R1, CD94, NKp80, CD16, and CD57, and the red lines underline the surface antigen changes that define each stage transition. Although not depicted, it is noted that CD56 expression is first detected at stage 2b (heterogeneous), peaks at stage 4b (CD56^bright^), and then decreases to the level of most peripheral blood NK cells at stage 6 (CD56^dim^). Also not depicted is killer immunoglobulin-like receptor expression, which is first detected within stage 4b in SLTs (40).

Human NK cells were originally thought to develop strictly within the bone marrow (BM) (3, 35). This notion was supported by the observation that Lin^−^CD56^+^ cytotoxic NK cells can be generated *in vitro* following culture of purified human BM CD34^+^ HPCs with either BM-derived stroma or with IL-15, which can be produced by stroma (36, 37). Nonetheless, more recent extensive *ex vivo* characterization of HPCs and putative downstream NK cell developmental intermediates (NKDIs) reveals that the latter are naturally enriched in SLTs, including tonsils, spleen, and LNs, suggesting that in humans NK cells can also, if not preferentially, develop in SLTs (Figure 1A) (38–42). Similar NKDIs have also been identified in the thymus, liver, and uterus (43–45). Thus, human NK cell development is likely not restricted to SLTs (46).

In 2006, five putative stages of human SLT NK cell development were described according to the differential expression of CD34, CD117, CD94, and CD16 (41, 47, 48). Stage 1 cells (Lin^−^CD34^+^CD117^−^CD94^−^CD16^−^) lack expression of the common IL-2/IL-15 receptor beta chain (IL-2/15Rβ, CD122) and are thus not responsive to exogenous soluble IL-2 or IL-15 *ex vivo*. However, they can generate NK cells when cultured in IL-15 plus other cytokines, such as Flt3 ligand and c-Kit ligand (KL) that likely induce CD122 expression and hence IL-15 responsiveness (49). In contrast, stage 2 cells (Lin^−^CD34^+^CD117^+^CD94^−^CD16^−^) constitutively express CD122 (albeit below the level of detection by flow cytometry) and can generate functionally mature NK cells *in vitro* in the presence of exogenous soluble IL-15 in media without other cytokines or support cells (41). Stage 2 cells also constitutively express a functional high affinity IL-2 receptor, including the IL-2Rα subunit (CD25), and can differentiate in response to picomolar concentrations of IL-2 *in vitro* (39). The physiologic relevance of this cytokine receptor expression is not yet known and has not been tested *in vivo*. One likely possibility is that these HPCs, which are naturally enriched and reside within the parafollicular T cell-rich regions of SLTs, can respond to T cell-derived IL-2 and differentiate into NK cells following T cell activation *in vivo* (39).

When originally tested in bulk polyclonal cultures under supportive *in vitro* conditions, stage 1 and stage 2 cells were multipotent and could give rise to T cells and DCs as well as to NK cells, although they could not generate B cells or myeloid cells (41). In contrast, human stage 3 cells (Lin^−^CD34^−^CD117^+^CD94^−^CD16^−^) lacked T cell and DC developmental potential. Stage 3 cells could, however, give rise to mature NK cells *in vitro* and *in vivo* and were thus originally proposed to represent committed NK cell precursors (41). Stage 3 cells are distinct from mature NK cells in that they lack high expression of T-BET and EOMES, cannot produce IFN-γ, and are incapable of mediating perforin-dependent cytotoxicity against MHC class I^−^ target cells. In contrast, these features are detected within the stage 4 (CD34^−^CD117^+/−^CD94^+^CD16^−^) and stage 5 (CD34^−^CD117^lo/−^CD94^+/−^CD16^+^) populations in SLTs (40, 41). Stage 5 cells are further distinguished by the constitutive expression of CD16, a low affinity receptor for the Fc portion of immunoglobulin, which provides for antibody-mediated cellular cytotoxicity (50).

## Recent Advances in Our Understanding of Human NKDIs

Continued *ex vivo* phenotypic and functional characterization of the aforementioned five putative NKDI populations in human SLTs has revealed a remarkable degree of heterogeneity within each stage. For example, in a study comparing tonsil- and thymus-derived HPCs, McClory et al. demonstrated that the human SLT stage 1 population, which expresses CD45RA and CD10, contains a minute subset of CD1a^+^CD11c^−^ cells that gives rise to T cells *ex vivo* and that shows substantial phenotypic overlap with CD34^+^CD45RA^+^CD10^+^CD1a^+^ T cell precursors in the thymus (51). McClory et al. were also able to trace a full putative pathway of tonsil T cell development branching directly from the tonsil CD34^+^CD45RA^+^CD10^+^CD1a^+^CD11c^−^ stage 1 subset and closely paralleling T cell development in the thymus. These data suggest that the developmental pathways of other lymphoid subsets overlap/intersect with the originally characterized NK cell developmental pathway in SLTs. Consistent with this notion, Montaldo et al. recently showed that human SLT-derived stage 2 cells constitutively express RORγt and can give rise to RORγt^+^CD117^+^ ILC3s (52). In that study, Montaldo et al. observed relatively low NK cell production from stage 2 cells under the *in vitro* conditions tested. Given those findings, the investigators concluded that the stage 2 cells represent lineage-specified ILC3 progenitors. However, Montaldo et al. did not assess for the capacity of stage 2 cells to differentiate into other lineages such as DCs and T cells, and as mentioned earlier, Freud et al. demonstrated that stage 2 cells can differentiate into T cells, DCs, and NK cells under supportive conditions (41).

In a subsequent study, Scoville et al. characterized two functionally distinct subsets of SLT stage 2 cells according to surface expression of the IL-1β receptor, IL-1R1 (42). Both IL-1R1^−^ (i.e., stage “2a”) and IL-1R1^+^ (i.e., stage “2b”) subsets express CD45RA, integrin β7, and *ID2*, and they show low or undetectable expression of CD10. However, the stage 2b cells are unique in their near uniform expression of the pan-ILC marker, CD161, their lack of detectable expression of *RAG1* mRNA, which is expressed in stage 1 and stage 2a cells, and their natural restriction to SLTs. In a series of experiments in which stage 1, stage 2a, and stage 2b cells were freshly purified and then cultured *in vitro* or transplanted into non-obese diabetic (NOD)-scid IL2Rgamma^null^ (NSG) immunodeficient mice treated with human IL-15, the stage 2b population was shown to be capable of giving rise to all four major subsets of ILCs (ILC1s, ILC2s, ILC3s, and NK cells), yet, they lacked T cell and DC developmental potential. In contrast, stage 1 and stage 2a cells could give rise to all ILC subsets as well as to T cells and DCs under the conditions tested (42). Thus, stage 2b cells appear to represent common ILC progenitors (CILPs) in humans.

Following the original discovery of SLT stage 3 cells (41), it was determined that cells within this population (Lin^−^CD34^−^CD117^+^CD94^−^CD16^−^) express AHR, CD127, RORγt, IL-1R1, and IL-22 (14, 53–55). According to the 2013 ILC classification, these features denote Group 3 ILCs (2). Thus, it is not yet clear if stage 3 cells and ILC3s are entirely overlapping in their phenotypic characteristics, and this is a subject of ongoing investigation (see below) (56). Within the stage 3/ILC3 population in human SLTs, there is marked heterogeneity with regards to the expression of numerous surface markers, including CD7, CD56, CD62L, HLA-DR, and NKp44 (14, 41, 57, 58). The significance of this heterogeneity is largely unknown, although it was shown that NKp44 expression closely correlates with IL-22 production *ex vivo* (53, 58).

Last, in a recent study by Freud and colleagues, the SLT stage 4 population, defined as Lin^−^CD34^−^CD117^+/−^CD94^+^CD16^−^, was shown to contain two functionally distinct subsets according to expression of the surface activating C-type lectin-like receptor, NKp80 (40), which is expressed on most if not all PB NK cells in healthy humans (59). Freud et al. described these SLT stage 4 subsets as stage “4a” (NKp80^−^) and stage “4b” (NKp80^+^), and they demonstrated that only the stage 4b subset is capable of IFN-γ production and perforin-dependent cellular cytotoxicity *ex vivo* (40). Consistent with these functional data, the surface expression of NKp80 among total SLT ILCs closely correlates with intracellular expression of T-BET, EOMES, and perforin. In contrast to stage 4b cells, stage 4a cells, which express CD94/NKG2A, lack the aforementioned functional and phenotypic features associated with mature NK cells and rather show a stage 3/ILC3-like profile including expression of CD117, CD127, IL-1R1, AHR, RORγt, and IL-22 (40). Following co-culture with allogeneic monocyte-derived DCs or transplantation into NSG mice treated with IL-15, purified SLT stage 4a cells gave rise to functional NK cells including some that were NKp80^+^CD16^+^ and coexpressed KIRs and CD57, the latter of which has been associated with terminal maturation and may represent a putative “stage 6” of human NK cell development (60, 61). Thus, stage 4a cells appear to comprise a naturally occurring and physiologic developmental intermediate population of cells emerging from stage 3 cells and giving rise to stage 4b cells. This hypothesis is supported by *ex vivo* patterns of surface antigen expression among total Lin^−^ ILCs in human SLTs (Figure 1B).

## Modeling Human NK Cell Development in the Context and Era of ILCs

Collectively, these recently published data described above support an updated linear model of human NK cell development (Figure 1C), which incorporates SLT NKDIs as well as the putative progressive development of human PB NK cells from CD56^bright^CD94^+^NKp80^+^CD16^−^CD57^−^ (i.e., stage 4b) to CD56^dim^CD94^+/−^NKp80^+^CD16^+^CD57^−^ (i.e., stage 5) to CD56^dim^CD94^+/−^NKp80^+^CD16^+^CD57^+^ (i.e., stage 6). Although this is still somewhat controversial, a linear developmental relationship between PB CD56^bright^ and CD56^dim^ NK cell subsets is supported by the recent detection of PB NK cell populations that appear to represent naturally occurring NKDI spanning the developmental continuum between CD56^bright^ and CD56^dim^ NK cells (27, 29). In addition, other studies have shown that CD56^bright^ NK cells can give rise to CD56^dim^ NK cells *in vitro* and *in vivo* (19, 24, 26).

Despite the accumulating evidence in support of this proposed model, it is likely that other pathways of human NK cell development exist *in vivo* and potentially account for the marked diversity among tissue-resident NK cells (62–64). For example, Renoux et al. recently described a putative lineage committed NK cell progenitor (NKP) population in human BM, blood, and SLTs characterized as Lin^–^CD34^+^CD38^+^CD123^–^CD45RA^+^CD7^+^CD10^+^CD127^−^ and that could only give rise to NK cells *in vitro* and *in vivo* (65). Given that this NKP is only partially CD117^+^ and reportedly lacks detectable expression of CD161 and CD127, it appears to be distinct from the aforementioned SLT CILP (i.e., stage 2b) population that is CD117^+^CD161^+^CD127^+^ and that can generate all ILCs including NK cells (42). Rather, the immunophenotype of the NKP described in the study by Renoux et al. appears to overlap with that of the stage 1 and stage 2a populations described above. Indeed, it will be important in future studies to determine how these various HPC populations are related. Regardless, the study by Renoux et al. raises the intriguing possibility of other NK cell developmental pathways in humans.

**Figure 2:**
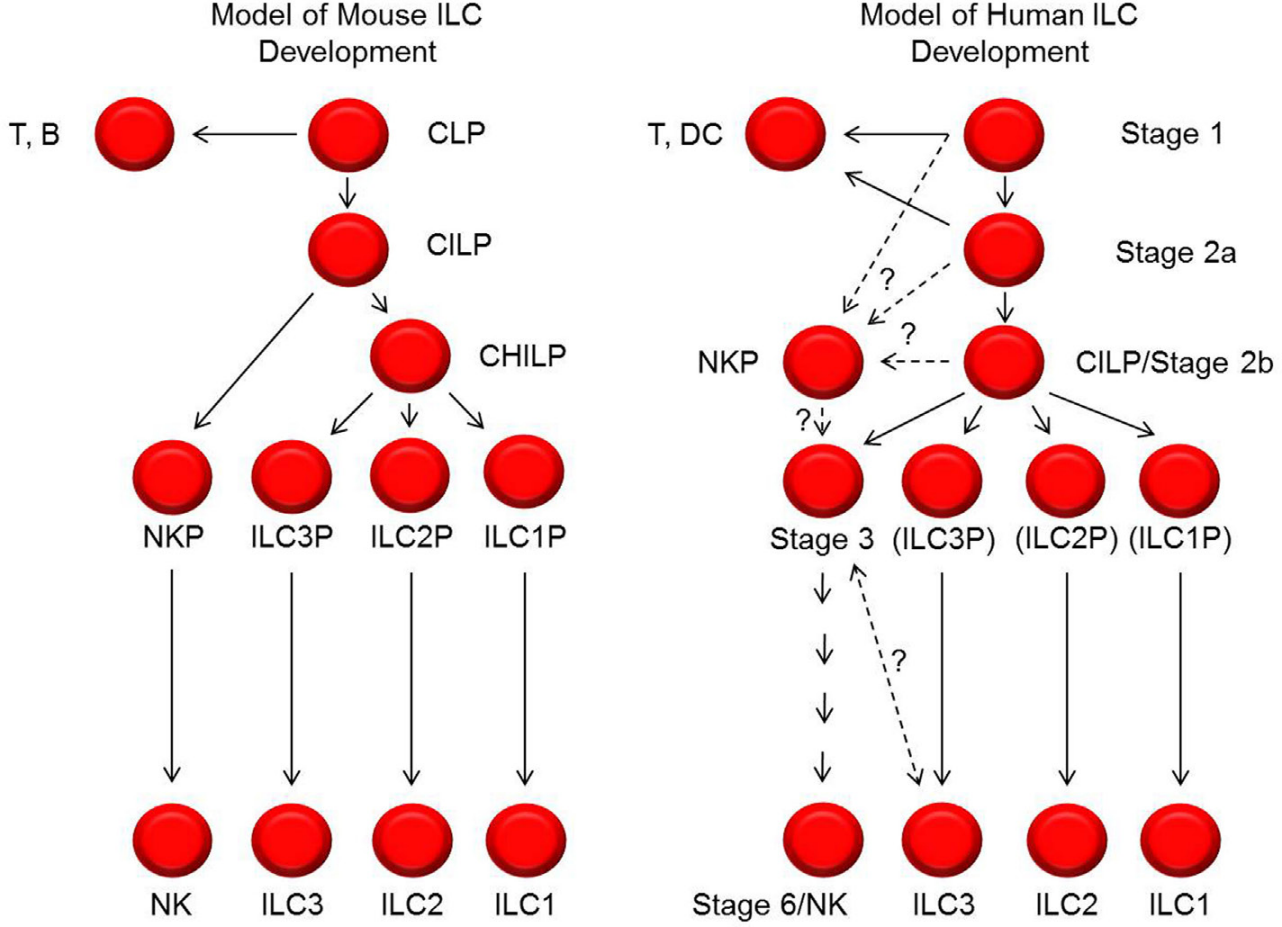
**Models of mouse and human innate lymphoid cell (ILC) development**. Shown on the left and right are schematic representations of the cellular intermediates and developmental pathways of mouse and human ILCs, respectively. The black lines represent progressive steps of differentiation and maturation that are currently supported by published data. The dashed lines represent possible steps and relationships of differentiation and maturation, but these have not yet been tested or definitively established. In particular, the relationships between stage 1, 2a, 2b, and 3 cells with NK cell progenitors (NKPs) described by Renoux et al. (65) are not yet known. In addition, the relationship and possible distinction between human stage 3 natural killer (NK) cell precursors and ILC3s is also not yet clear. In the diagram to the right depicting human ILC development, the labels ILC3P (for ILC3 progenitor), ILC2P (for ILC2 progenitor), and ILC1P (for ILC1 progenitor) are shown in parentheses to convey that these are theoretical populations that have not been identified. CILP, common innate lymphoid progenitor; CHILP, common helper innate lymphoid progenitor.

It is also important to note that the human model of NK cell development depicted here differs from contemporary models of murine NK cell development (Figure 2) (1, 7, 12). Aside from inherent challenges in comparing these developmental processes due to differences in species-specific antigen expression, there may be fundamental differences between the species with regards to the developmental relationship(s) between NK cells and other ILCs and with regards to transcription factor expression patterns. For example, in mice, all ILC subsets are thought to derive from a CILP population that subsequently gives rise to committed NKPs as well as to common helper ILC progenitor cells (CHILPs) that can differentiate into ILC1s, ILC2s, and ILC3s, but not NK cells (66–70). While putative human CILPs (SLT stage 2b cells) and NKPs have been described as discussed above (42, 65), it is not yet clear if these two populations are related in the same way that the analogous populations in mice have been described. In addition, a human CHILP population has not been identified.

Another potential difference between mouse and human NK cell development relates to the relationship between NK cells and ILC3s. In mice, these two ILC subsets appear to be developmentally distinct (6), because in addition to these CHILP data described above, genetic fate-mapping (fm) studies for *Rorc2*, which encodes Rorγt, showed that mouse ILC3s are *Rorc2* fm^+^ whereas mouse Eomes^+^ NK cells are *Rorc2* fm^−^ (68, 71, 72). In contrast, Scoville et al. recently demonstrated that the putative human CILP (SLT stage 2b) population expresses RORγt and that human PB CD56^bright^ NK cells, which are NKp80^+^EOMES^+^ (4, 40, 59, 73, 74), constitutively express detectable *RORC2* transcript (42). Thus, as opposed to mouse NK cells, at least some human NK cells would be fm^+^ for *RORC2* if such an experiment could be performed. In addition, as mentioned earlier, there is substantial phenotypic overlap between cells originally described as “stage 3” NK cell precursors and cells denoted as “ILC3s” by other groups, raising the question as to whether or not these cell types are different or comprise the same population in humans (56). To address this issue, Crellin et al. previously evaluated the *in vitro* differentiation potentials of Lin^−^CD34^−^CD161^+^CD117^+^ ILCs according to the differential expression of CD127 (75). They demonstrated that a minute CD127^−^ fraction preferentially gave rise to NK cells whereas the predominant CD127^+^ fraction mostly retained ILC3 features. Thus CD127 has since been touted as a critical marker distinguishing human stage 3 NK cell precursors from ILC3s (1, 6). However, in the study by Crellin et al., CD94^+^ NK cells, which can express CD117 and CD161 (18, 76), were not excluded in the pre-culture sorting preparations (75). Moreover, other reports suggest that the *in vitro* conditions used by Crellin et al. may be better optimized for supporting NK cell differentiation; conditions such as co-culture with OP9-DL1 stroma or DCs may be needed (40, 42). As such, the results provided in the Crellin et al. study could be due at least in part to the culture conditions employed and/or the preferential expansion of CD94^+^ NK cells that were present in the sorted Lin^−^CD34^−^CD161^+^CD117^+^CD127^−^ fractions. Last, we note that CD127 is constitutively expressed on PB CD56^bright^ NK cells (19, 20), and so excluding any CD127^+^ cells as potential NK cell precursors may be incorrect. Thus, to the best of our knowledge, there is as yet no reliable immunophenotypic strategy to distinguish human stage 3 NK cell precursors from ILC3s. Further investigation is needed.

## Concluding Remarks and Future Directions

The goals of investigating human NK cell development are to understand malignant counterparts and to facilitate the design and implementation of optimal immunotherapies for patients with cancer and potentially other diseases. Much progress has been made; however, the recent discovery of non-NK ILCs has required a reassessment of models of NK cell development in both mice and humans. More work is also needed in order to understand how all ILCs develop and to determine if the putative pathway of human NK cell development in SLTs is representative of NK cell development in other tissues.

## Author Contributions

SS, AF, and MC conceived the idea and wrote the manuscript.

## Conflict of Interest Statement

The authors declare that the research was conducted in the absence of any commercial or financial relationships that could be construed as a potential conflict of interest.

## References

[1] Artis D Spits H. The biology of innate lymphoid cells. Nature (2015) 517(7534):293–301. 10.1038/nature14189 25592534

[2] Spits H Artis D Colonna M Diefenbach A Di Santo JP Eberl G Innate lymphoid cells – a proposal for uniform nomenclature. Nat Rev Immunol (2013) 13(2):145–9. 10.1038/nri3365 23348417

[3] Caligiuri MA. Human natural killer cells. Blood (2008) 112(3):461–9. 10.1182/blood-2007-09-077438 18650461 PMC2481557

[4] Spits H Bernink JH Lanier L. NK cells and type 1 innate lymphoid cells: partners in host defense. Nat Immunol (2016) 17(7):758–64. 10.1038/ni.3482 27328005

[5] Kim BS Artis D. Group 2 innate lymphoid cells in health and disease. Cold Spring Harb Perspect Biol (2015) 7(5):a016337. 10.1101/cshperspect.a016337 25573713 PMC4448620

[6] Montaldo E Juelke K Romagnani C. Group 3 innate lymphoid cells (ILC3s): origin, differentiation, and plasticity in humans and mice. Eur J Immunol (2015) 45(8):2171–82. 10.1002/eji.201545598 26031799

[7] Diefenbach A Colonna M Koyasu S. Development, differentiation, and diversity of innate lymphoid cells. Immunity (2014) 41(3):354–65. 10.1016/j.immuni.2014.09.005 25238093 PMC4171710

[8] Eberl G Colonna M Di Santo JP McKenzie AN Innate lymphoid cells. Innate lymphoid cells: a new paradigm in immunology. Science (2015) 348(6237):aaa6566 10.1126/science.aaa6566 25999512 PMC5658207

[9] Eberl G Di Santo JP Vivier E The brave new world of innate lymphoid cells. Nat Immunol (2015) 16(1):1–5. 10.1038/ni.3059 25521670

[10] Hazenberg MD Spits H. Human innate lymphoid cells. Blood (2014) 124(5):700–9. 10.1182/blood-2013-11-427781 24778151

[11] Klose CS Artis D Innate lymphoid cells as regulators of immunity, inflammation and tissue homeostasis. Nat Immunol (2016) 17(7):765–74. 10.1038/ni.3489 27328006

[12] Zook EC Kee BL. Development of innate lymphoid cells. Nat Immunol (2016) 17(7):775–82. 10.1038/ni.3481 27328007

[13] Bernink JH Peters CP Munneke M te Velde AA Meijer SL Weijer K Human type 1 innate lymphoid cells accumulate in inflamed mucosal tissues. Nat Immunol (2013) 14(3):221–9. 10.1038/ni.2534 23334791

[14] Cupedo T Crellin NK Papazian N Rombouts EJ Weijer K Grogan JL Human fetal lymphoid tissue-inducer cells are interleukin 17-producing precursors to RORC+ CD127+ natural killer-like cells. Nat Immunol (2009) 10(1):66–74. 10.1038/ni.1668 19029905

[15] Fuchs A Vermi W Lee JS Lonardi S Gilfillan S Newberry RD Intraepithelial type 1 innate lymphoid cells are a unique subset of IL-12- and IL-15-responsive IFN-γ-producing cells. Immunity (2013) 38(4):769–81. 10.1016/j.immuni.2013.02.010 23453631 PMC3634355

[16] Mjösberg JM Trifari S Crellin NK Peters CP van Drunen CM Piet B Human IL-25- and IL-33-responsive type 2 innate lymphoid cells are defined by expression of CRTH2 and CD161. Nat Immunol (2011) 12(11):1055–62. 10.1038/ni.2104 21909091

[17] Cortez VS Robinette ML Colonna M. Innate lymphoid cells: new insights into function and development. Curr Opin Immunol (2015) 32:71–7. 10.1016/j.coi.2015.01.004 25615701 PMC4648536

[18] Azzoni L Zatsepina O Abebe B Bennett IM Kanakaraj P Perussia B. Differential transcriptional regulation of CD161 and a novel gene, 197/15a, by IL-2, IL-15, and IL-12 in NK and T cells. J Immunol (1998) 161(7):3493–500. 9759869

[19] Romagnani C Juelke K Falco M Morandi B D’Agostino A Costa R CD56brightCD16- killer Ig-like receptor- NK cells display longer telomeres and acquire features of CD56dim NK cells upon activation. J Immunol (2007) 178(8):4947–55. 10.4049/jimmunol.178.8.4947 17404276

[20] Vosshenrich CA García-Ojeda ME Samson-Villéger SI Pasqualetto V Enault L Richard-Le Goff O A thymic pathway of mouse natural killer cell development characterized by expression of GATA-3 and CD127. Nat Immunol (2006) 7(11):1217–24. 10.1038/ni1395 17013389

[21] Moretta L Montaldo E Vacca P Del Zotto G Moretta F Merli P Human natural killer cells: origin, receptors, function, and clinical applications. Int Arch Allergy Immunol (2014) 164(4):253–64. 10.1159/000365632 25323661

[22] Cooper MA Fehniger TA Caligiuri MA. The biology of human natural killer-cell subsets. Trends Immunol (2001) 22(11):633–40. 10.1016/S1471-4906(01)02060-9 11698225

[23] Lanier LL Le AM Civin CI Loken MR Phillips JH. The relationship of CD16 (Leu-11) and Leu-19 (NKH-1) antigen expression on human peripheral blood NK cells and cytotoxic T lymphocytes. J Immunol (1986) 136(12):4480–6. 3086432

[24] Chan A Hong DL Atzberger A Kollnberger S Filer AD Buckley CD CD56bright human NK cells differentiate into CD56dim cells: role of contact with peripheral fibroblasts. J Immunol (2007) 179(1):89–94. 10.4049/jimmunol.179.1.89 17579025

[25] Dulphy N Haas P Busson M Belhadj S Peffault de Latour R Robin M An unusual CD56(bright) CD16(low) NK cell subset dominates the early posttransplant period following HLA-matched hematopoietic stem cell transplantation. J Immunol (2008) 181(3):2227–37. 10.4049/jimmunol.181.3.2227 18641363

[26] Huntington ND Legrand N Alves NL Jaron B Weijer K Plet A IL-15 trans-presentation promotes human NK cell development and differentiation in vivo. J Exp Med (2009) 206(1):25–34. 10.1084/jem.20082013 19103877 PMC2626663

[27] Juelke K Killig M Luetke-Eversloh M Parente E Gruen J Morandi B CD62L expression identifies a unique subset of polyfunctional CD56dim NK cells. Blood (2010) 116(8):1299–307. 10.1182/blood-2009-11-253286 20505160

[28] Ouyang Q Baerlocher G Vulto I Lansdorp PM. Telomere length in human natural killer cell subsets. Ann N Y Acad Sci (2007) 1106:240–52. 10.1196/annals.1392.001 17303822

[29] Yu J Mao HC Wei M Hughes T Zhang J Park IK CD94 surface density identifies a functional intermediary between the CD56bright and CD56dim human NK-cell subsets. Blood (2010) 115(2):274–81. 10.1182/blood-2009-04-215491 19897577 PMC2808153

[30] Grzywacz B Kataria N Kataria N Blazar BR Miller JS Verneris MR. Natural killer-cell differentiation by myeloid progenitors. Blood (2011) 117(13):3548–58. 10.1182/blood-2010-04-281394 21173117 PMC3072878

[31] Mailliard RB Alber SM Shen H Watkins SC Kirkwood JM Herberman RB IL-18-induced CD83+CCR7+ NK helper cells. J Exp Med (2005) 202(7):941–53. 10.1084/jem.20050128 16203865 PMC2213172

[32] Perussia B Chen Y Loza MJ. Peripheral NK cell phenotypes: multiple changing of faces of an adapting, developing cell. Mol Immunol (2005) 42(4):385–95. 10.1016/j.molimm.2004.07.017 15607789

[33] Vukicevic M Chalandon Y Helg C Matthes T Dantin C Huard B CD56bright NK cells after hematopoietic stem cell transplantation are activated mature NK cells that expand in patients with low numbers of T cells. Eur J Immunol (2010) 40(11):3246–54. 10.1002/eji.200940016 20957748

[34] Wu C Li B Lu R Koelle SJ Yang Y Jares A Clonal tracking of rhesus macaque hematopoiesis highlights a distinct lineage origin for natural killer cells. Cell Stem Cell (2014) 14(4):486–99. 10.1016/j.stem.2014.01.020 24702997 PMC3979461

[35] Colucci F Caligiuri MA Di Santo JP. What does it take to make a natural killer? Nat Rev Immunol (2003) 3(5):413–25. 10.1038/nri1088 12766763

[36] Miller JS Alley KA McGlave P. Differentiation of natural killer (NK) cells from human primitive marrow progenitors in a stroma-based long-term culture system: identification of a CD34+7+ NK progenitor. Blood (1994) 83(9):2594–601. 7513206

[37] Mrozek E Anderson P Caligiuri MA. Role of interleukin-15 in the development of human CD56+ natural killer cells from CD34+ hematopoietic progenitor cells. Blood (1996) 87(7):2632–40. 8639878

[38] Eissens DN Spanholtz J van der Meer A van Cranenbroek B Dolstra H Kwekkeboom J Defining early human NK cell developmental stages in primary and secondary lymphoid tissues. PLoS One (2012) 7(2):e30930. 10.1371/journal.pone.0030930 22319595 PMC3272048

[39] Freud AG Becknell B Roychowdhury S Mao HC Ferketich AK Nuovo GJ A human CD34(+) subset resides in lymph nodes and differentiates into CD56bright natural killer cells. Immunity (2005) 22(3):295–304. 10.1016/j.immuni.2005.01.013 15780987

[40] Freud AG Keller KA Scoville SD Mundy-Bosse BL Cheng S Youssef Y NKp80 defines a critical step during human natural killer cell development. Cell Rep (2016) 16(2):379–91. 10.1016/j.celrep.2016.05.095 27373165 PMC4970225

[41] Freud AG Yokohama A Becknell B Lee MT Mao HC Ferketich AK Evidence for discrete stages of human natural killer cell differentiation in vivo. J Exp Med (2006) 203(4):1033–43. 10.1084/jem.20052507 16606675 PMC2118285

[42] Scoville SD Mundy-Bosse BL Zhang MH Chen L Zhang X Keller KA A progenitor cell expressing transcription factor RORgammat generates all human innate lymphoid cell subsets. Immunity (2016) 44(5):1140–50. 10.1016/j.immuni.2016.04.007 27178467 PMC4893782

[43] Hidalgo L Martinez VG Valencia J Hernandez-Lopez C Vazquez MN Nunez JR Expression of BMPRIA on human thymic NK cell precursors: role of BMP signaling in intrathymic NK cell development. Blood (2012) 119(8):1861–71. 10.1182/blood-2011-07-370650 22210872

[44] Male V Hughes T McClory S Colucci F Caligiuri MA Moffett A. Immature NK cells, capable of producing IL-22, are present in human uterine mucosa. J Immunol (2010) 185(7):3913–8. 10.4049/jimmunol.1001637 20802153 PMC3795409

[45] Moroso V Famili F Papazian N Cupedo T van der Laan LJ Kazemier G NK cells can generate from precursors in the adult human liver. Eur J Immunol (2011) 41(11):3340–50. 10.1002/eji.201141760 21830211

[46] Yu J Freud AG Caligiuri MA. Location and cellular stages of natural killer cell development. Trends Immunol (2013) 34(12):573–82. 10.1016/j.it.2013.07.005 24055329 PMC3852183

[47] Freud AG Caligiuri MA. Human natural killer cell development. Immunol Rev (2006) 214:56–72. 10.1111/j.1600-065X.2006.00451.x 17100876

[48] Grzywacz B Kataria N Sikora M Oostendorp RA Dzierzak EA Blazar BR Coordinated acquisition of inhibitory and activating receptors and functional properties by developing human natural killer cells. Blood (2006) 108(12):3824–33. 10.1182/blood-2006-04-020198 16902150 PMC1895469

[49] Yu H Fehniger TA Fuchshuber P Thiel KS Vivier E Carson WE Flt3 ligand promotes the generation of a distinct CD34(+) human natural killer cell progenitor that responds to interleukin-15. Blood (1998) 92(10):3647–57. 9808558

[50] Lanier LL Le AM Phillips JH Warner NL Babcock GF. Subpopulations of human natural killer cells defined by expression of the Leu-7 (HNK-1) and Leu-11 (NK-15) antigens. J Immunol (1983) 131(4):1789–96. 6225799

[51] McClory S Hughes T Freud AG Briercheck EL Martin C Trimboli AJ Evidence for a stepwise program of extrathymic T cell development within the human tonsil. J Clin Invest (2012) 122(4):1403–15. 10.1172/JCI46125 22378041 PMC3314444

[52] Montaldo E Teixeira-Alves LG Glatzer T Durek P Stervbo U Hamann W Human RORγt(+)CD34(+) cells are lineage-specified progenitors of group 3 RORγt(+) innate lymphoid cells. Immunity (2014) 41(6):988–1000. 10.1016/j.immuni.2014.11.010 25500367

[53] Cella M Fuchs A Vermi W Facchetti F Otero K Lennerz JK A human natural killer cell subset provides an innate source of IL-22 for mucosal immunity. Nature (2009) 457(7230):722–5. 10.1038/nature07537 18978771 PMC3772687

[54] Hughes T Becknell B Freud AG McClory S Briercheck E Yu J Interleukin-1beta selectively expands and sustains interleukin-22+ immature human natural killer cells in secondary lymphoid tissue. Immunity (2010) 32(6):803–14. 10.1016/j.immuni.2010.06.007 20620944 PMC3742307

[55] Hughes T Becknell B McClory S Briercheck E Freud AG Zhang XL Stage 3 immature human natural killer cells found in secondary lymphoid tissue constitutively and selectively express the T(H)17 cytokine interleukin-22. Blood (2009) 113(17):4008–10. 10.1182/blood-2008-12-192443 19244159 PMC2673127

[56] Freud AG Yu J Caligiuri MA. Human natural killer cell development in secondary lymphoid tissues. Semin Immunol (2014) 26(2):132–7. 10.1016/j.smim.2014.02.008 24661538 PMC4010312

[57] Bjorklund AK Forkel M Picelli S Konya V Theorell J Friberg D The heterogeneity of human CD127(+) innate lymphoid cells revealed by single-cell RNA sequencing. Nat Immunol (2016) 17(4):451–60. 10.1038/ni.3368 26878113

[58] Hoorweg K Peters CP Cornelissen F Aparicio-Domingo P Papazian N Kazemier G Functional differences between human NKp44(-) and NKp44(+) RORC(+) innate lymphoid cells. Front Immunol (2012) 3:72. 10.3389/fimmu.2012.00072 22566953 PMC3342004

[59] Vitale M Falco M Castriconi R Parolini S Zambello R Semenzato G Identification of NKp80, a novel triggering molecule expressed by human NK cells. Eur J Immunol (2001) 31(1):233–42. 10.1002/1521-4141(200101)31:1<233::AID-IMMU233>3.0.CO;2-4 11265639

[60] Bjorkstrom NK Riese P Heuts F Andersson S Fauriat C Ivarsson MA Expression patterns of NKG2A, KIR, and CD57 define a process of CD56dim NK-cell differentiation uncoupled from NK-cell education. Blood (2010) 116(19):3853–64. 10.1182/blood-2010-04-281675 20696944

[61] Lopez-Verges S Milush JM Pandey S York VA Arakawa-Hoyt J Pircher H CD57 defines a functionally distinct population of mature NK cells in the human CD56dimCD16+ NK-cell subset. Blood (2010) 116(19):3865–74. 10.1182/blood-2010-04-282301 20733159 PMC2981540

[62] Lugthart G Melsen JE Vervat C van Ostaijen-Ten Dam MM Corver WE Roelen DL Human lymphoid tissues harbor a distinct CD69+CXCR6+ NK cell population. J Immunol (2016) 197(1):78–84. 10.4049/jimmunol.1502603 27226093

[63] Melsen JE Lugthart G Lankester AC Schilham MW. Human circulating and tissue-resident CD56(bright) natural killer cell populations. Front Immunol (2016) 7:262. 10.3389/fimmu.2016.00262 27446091 PMC4927633

[64] Sojka DK Plougastel-Douglas B Yang L Pak-Wittel MA Artyomov MN Ivanova Y Tissue-resident natural killer (NK) cells are cell lineages distinct from thymic and conventional splenic NK cells. Elife (2014) 3:e01659. 10.7554/eLife.01659 24714492 PMC3975579

[65] Renoux VM Zriwil A Peitzsch C Michaelsson J Friberg D Soneji S Identification of a human natural killer cell lineage-restricted progenitor in fetal and adult tissues. Immunity (2015) 43(2):394–407. 10.1016/j.immuni.2015.07.011 26287684

[66] Constantinides MG Gudjonson H McDonald BD Ishizuka IE Verhoef PA Dinner AR PLZF expression maps the early stages of ILC1 lineage development. Proc Natl Acad Sci U S A (2015) 112(16):5123–8. 10.1073/pnas.1423244112 25838284 PMC4413309

[67] Constantinides MG McDonald BD Verhoef PA Bendelac A. A committed precursor to innate lymphoid cells. Nature (2014) 508(7496):397–401. 10.1038/nature13047 24509713 PMC4003507

[68] Klose CS Flach M Möhle L Rogell L Hoyler T Ebert K Differentiation of type 1 ILCs from a common progenitor to all helper-like innate lymphoid cell lineages. Cell (2014) 157(2):340–56. 10.1016/j.cell.2014.03.030 24725403

[69] Yang Q Li F Harly C Xing S Ye L Xia X TCF-1 upregulation identifies early innate lymphoid progenitors in the bone marrow. Nat Immunol (2015) 16(10):1044–50. 10.1038/ni.3248 26280998 PMC4575643

[70] Yu X Wang Y Deng M Li Y Ruhn KA Zhang CC The basic leucine zipper transcription factor NFIL3 directs the development of a common innate lymphoid cell precursor. Elife (2014) 3:e04406 10.7554/eLife.04406 PMC4356142 25310240

[71] Satoh-Takayama N Lesjean-Pottier S Vieira P Sawa S Eberl G Vosshenrich CA IL-7 and IL-15 independently program the differentiation of intestinal CD3-NKp46+ cell subsets from Id2-dependent precursors. J Exp Med (2010) 207(2):273–80. 10.1084/jem.20092029 20142427 PMC2822619

[72] Vonarbourg C Mortha A Bui VL Hernandez PP Kiss EA Hoyler T Regulated expression of nuclear receptor RORγt confers distinct functional fates to NK cell receptor-expressing RORγt(+) innate lymphocytes. Immunity (2010) 33(5):736–51. 10.1016/j.immuni.2010.10.017 21093318 PMC3042726

[73] Knox JJ Cosma GL Betts MR McLane LM. Characterization of T-bet and eomes in peripheral human immune cells. Front Immunol (2014) 5:217. 10.3389/fimmu.2014.00217 24860576 PMC4030168

[74] Simonetta F Pradier A Roosnek E. T-bet and eomesodermin in NK cell development, maturation, and function. Front Immunol (2016) 7:241. 10.3389/fimmu.2016.00241 27379101 PMC4913100

[75] Crellin NK Trifari S Kaplan CD Cupedo T Spits H. Human NKp44+IL-22+ cells and LTi-like cells constitute a stable RORC+ lineage distinct from conventional natural killer cells. J Exp Med (2010) 207(2):281–90. 10.1084/jem.20091509 20142432 PMC2822607

[76] Matos ME Schnier GS Beecher MS Ashman LK William DE Caligiuri MA. Expression of a functional c-kit receptor on a subset of natural killer cells. J Exp Med (1993) 178(3):1079–84. 10.1084/jem.178.3.1079 7688785 PMC2191187

